# Antithrombotic Therapy Increases the Risk of Bleeding after Endoscopic Submucosal Dissection for Early Gastric Cancer: A Propensity Score-Matched Analysis

**DOI:** 10.3390/cancers15102844

**Published:** 2023-05-19

**Authors:** Tae-Se Kim, Byung-Hoon Min, Sun-Young Baek, Kyunga Kim, Yang-Won Min, Hyuk Lee, Jun-Haeng Lee, Poong-Lyul Rhee, Jae J. Kim

**Affiliations:** 1 Department of Medicine, Samsung Medical Center, Sungkyunkwan University School of Medicine, 81 Irwon-ro, Gangnam-gu, Seoul 06351, Republic of Korea; tsk1029@naver.com (T.-S.K.);; 2 Biomedical Statistics Center, Data Science Research Institute, Research Institute for Future Medicine, Samsung Medical Center, Seoul 06351, Republic of Koreakyunga.j.kim@samsung.com (K.K.); 3 Department of Digital Health, Samsung Advanced Institute for Health Sciences and Technology, Sungkyunkwan University, Seoul 06355, Republic of Korea

**Keywords:** anticoagulants, antiplatelets, endoscopic submucosal dissection, postoperative hemorrhage

## Abstract

**Simple Summary:**

Endoscopic submucosal dissection (ESD) for early gastric cancer patients using antithrombotic agents (ATA) is becoming more common than ever before due to the aging of society and rising ATA prescription rates. The relationship between perioperative ATA use and post-ESD bleeding is still debatable at this time. Furthermore, it is uncertain whether the suggested duration of ATA cessation in current guidelines is sufficient to normalize the bleeding risk. This study examined the effects of ATA on post-ESD bleeding through propensity score-matching analysis between ATA users and non-users. We discovered that ATA use was independently associated with a higher incidence of post-ESD bleeding. This tendency was significantly observed in patients who either continued or insufficiently discontinued ATA and those who sufficiently discontinued ATA, according to current guidelines. Given these findings, we suggest careful observation after ESD for patients using ATA, regardless of their cessation status.

**Abstract:**

Whether antithrombotic agent (ATA) usage increases the risk of gastric post-endoscopic submucosal dissection (ESD) bleeding remains controversial. The aim of this study was to elucidate the effects of usage, type, and cessation timing of ATA on post-ESD bleeding. A total of 4775 early gastric cancer patients undergoing ESD were analyzed; 1:3 propensity score matching between ATA and non-ATA groups resulted in 318 and 767 matched patients in each group, respectively. Outcomes were compared between the two groups using a generalized estimating equation method. After matching, post-ESD bleeding rates in ATA users and non-users were 9.1% and 4.2%, respectively (*p* = 0.001). In multivariable analysis, ATA usage was independently associated with an increased risk of post-ESD bleeding (adjusted odds ratio: 2.28, 95% confidence interval: 1.34–3.86). Both the continued or insufficient cessation groups and the sufficient cessation group had an increased incidence of post-ESD bleeding compared to their matched controls (12.5% versus 5.2%, *p* = 0.048; 8.1% versus 3.9%, *p* = 0.014). Post-ESD bleeding rates in antiplatelet agent users were significantly higher than those of their matched controls (8.3% versus 4.2%, *p* = 0.010). ATA usage increased the risk of post-ESD bleeding even after its sufficient cessation. Careful observation after ESD is required regardless of the cessation status of ATA.

## 1. Introduction

Endoscopic submucosal dissection (ESD) is accepted as an effective treatment for early gastric cancer (EGC) without metastases with a 10-year overall survival rate exceeding 96% [1]. Despite recent advances in ESD techniques, post-ESD bleeding remains problematic; it can be life-threatening [2] or require emergency surgery [3]. Previous studies have reported several risk factors for bleeding after gastric ESD including size of resected specimen, [3,4,5], comorbidities, [6,7], and antithrombotic therapy [8,9,10].

With an aging society, the number of EGC patients taking antithrombotic agents (ATA) (antiplatelet agent (AP) and anticoagulant agent (AC)) has greatly increased and the perioperative management of ATA before and after ESD has become a matter of great concern. Previous studies on the perioperative management of ATA have been limited in that most studies were small and retrospective, without a control group of non-ATA users, which made these studies vulnerable to confounding biases. Due to these limitations, whether perioperative usage of ATA increases the risk of post-ESD bleeding in patients undergoing gastric ESD remains controversial. Although Cho et al. [11] argued that the continuous use of aspirin can increase the risk of post-ESD bleeding, several previous studies have reported contrary results [12,13,14]. In addition, the effects of the type of ATA and timing of ATA cessation on post-ESD bleeding remain unclear. With these controversies, recommendations from recent guidelines regarding the perioperative usage of ATA are inconsistent. Asian Pacific guidelines classify ESD as an ultra-high-risk procedure for bleeding and recommend discontinuing all ATAs including aspirin prior to ESD [15]. However, European and American guidelines recommend continuing aspirin before all endoscopic procedures including ESD [16,17].

Recently, a Japanese nationwide multicenter propensity score analysis [18] showed that antithrombotic therapy increased the incidence of post-ESD bleeding in those who either continued or discontinued ATA. However, their study did not account for the duration of ATA cessation. So et al. [19] conducted a propensity score matching analysis that considered the duration of ATA cessation. However, their study was limited in that the matching variables were limited in number (n = 7) and artificially chosen. A meta-analysis by Dong et al. [10] also found that ATA usage was associated with post-ESD bleeding, regardless of whether the drug was continued or discontinued. However, their study was limited due to significant heterogeneity among the included studies. In the present study, we conducted a propensity score-matching analysis on the effect of antithrombotic therapy on post-ESD bleeding using extensive clinical variables (n = 13) based on a group comparison between ATA users and non-users to minimize the influence of confounding biases. We also examined whether the type of ATA and the timing of ATA cessation were associated with the risk of post-ESD bleeding in ATA users in comparison with their matched controls.

## 2. Materials and Methods

### 2.1. Patients

**Figure 1:**
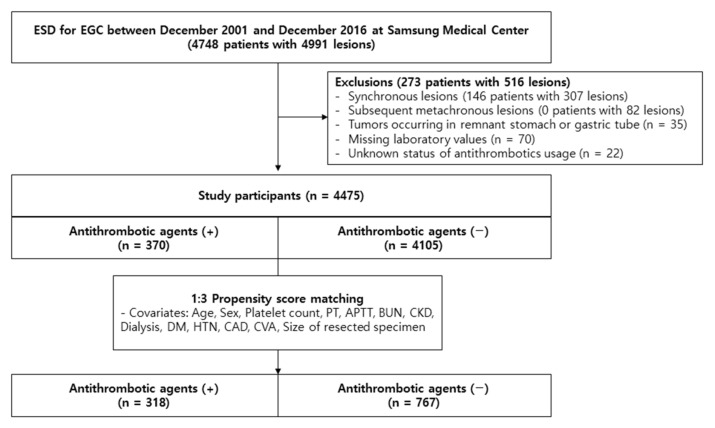
Flowchart showing the selection of patients for this study. ESD, endoscopic submucosal dissection; EGC, early gastric cancer; PT, prothrombin time; APTT, activated partial thromboplastin time; BUN, blood urea nitrogen; CKD, chronic kidney disease; DM, diabetes mellitus; HTN, hypertension; CAD, coronary artery disease; CVA, cerebrovascular accident.

At Samsung Medical Center, 4748 patients with 4991 EGC lesions underwent ESD between December 2001 and December 2016. A total of 146 patients with 307 synchronous lesions were excluded from this group of patients. Only the first case of each patient’s metachronous lesion was included; all subsequent cases were omitted (82 cases). Of the remaining, we excluded 35 patients with 35 lesions developing in the remnant stomach or gastric tube after esophagectomy. A total of 22 patients with uncertain ATA usage status and a total of 70 patients with missing laboratory values were also excluded. Finally, 4475 patients with 4475 single lesions that had undergone ESD treatment were included in the analysis. Among them, 370 patients had been exposed to ATA whereas 4105 patients had not. After 1:3 propensity score matching, 318 patients who were exposed to ATA before ESD and their 767 matched controls without ATA exposure were evaluated (Figure 1). The Institutional Review Board (IRB) of Samsung Medical Center approved this study (approval number: SMC 2022-07-191). Considering the retrospective nature of this study, informed patient consent was waived by the IRB. This research was conducted in accordance with the guidelines of the Declaration of Helsinki.

From an intranet database of Samsung Medical Center, the following clinicopathological factors characterizing patient status at the time of ESD were extracted: ATA usage and cessation status, age, sex, platelet count, prothrombin time international normalized ratio (PT INR), activated partial thromboplastin time (APTT), blood urea nitrogen (BUN), presence of comorbidities (such as chronic kidney disease (CKD), dialysis, diabetes mellitus (DM), hypertension (HTN), coronary artery disease (CAD), cerebrovascular accident (CVA), and liver cirrhosis (LC)), size of the resected specimen, endoscopic tumor morphology, and tumor location. CKD was defined as a decreased estimated glomerular filtration rate of less than 60 mL/min/1.73 m2, which corresponded to stages 3, 4, or 5 according to the K/DOQI CKD classification [20].

### 2.2. Outcomes

The primary outcome was post-ESD bleeding. Post-ESD bleeding was defined when one of the following criteria was met within 4 weeks after ESD: (1) the presence of clinical signs of bleeding (hematemesis, melena, or hematochezia) that required endoscopic, radiologic, or surgical intervention for bleeding from the ESD site or (2) a decrease of more than 2 g/dL of blood hemoglobin, which was diagnosed as ESD site bleeding by endoscopy. Secondary outcomes were thromboembolic events after ESD.

### 2.3. ESD Procedure and Follow-Up after ESD

ESD was carried out by skilled endoscopists in a standard fashion. Clinicopathological evaluations and detailed descriptions of the ESD procedures used in our institution have been published elsewhere. [21,22]. All patients visited the outpatient clinic two weeks following discharge to check the final ESD pathology. During this visit, the occurrence of adverse events was also checked.

### 2.4. Antithrombotic Agents

AP included aspirin and clopidogrel. As there were no users among the study population, other P2Y12 receptor antagonists such as prasugrel and ticagrelor were excluded from the analysis. AC included warfarin and direct oral anticoagulants (DOAC: dabigatran, rivaroxaban, apixaban, and edoxaban). The decision to discontinue ATA generally followed current guidelines [15,16,17]. However, depending on the patient’s situation, case-by-case adjustments were made based on cardiologist or neurologist consultations.

According to the timing of ATA cessation, patients taking ATA were subdivided into two groups: a ‘sufficient cessation group’ and a ‘continued or insufficient cessation group’. Sufficient cessation was defined according to previous guidelines as follows: (1) discontinuing aspirin [11,12,13,19] or clopidogrel [16,17,23] at least 7 days before ESD, (2) discontinuing warfarin [15,16,17] at least 5 days before ESD with documentation of PT INR normalization, and (3) discontinuing DOAC [15,17] at least 2 days before ESD. In patients taking more than one ATA, patients were considered to belong to the sufficient cessation group only when all ATAs were stopped according to the above-mentioned criteria. Continued or insufficient cessation was acknowledged if patients continued taking ATA until the day of ESD or discontinued ATA before ESD for a shorter period than the sufficient cessation group. In cases of heparin bridging therapy (HBT), patients were considered to belong to the continued or insufficient cessation group because previous studies consistently reported increased bleeding risk associated with HBT [10,24,25].

### 2.5. Statistical Analysis

Before and after propensity score matching, the clinicopathological features of those who were taking ATA and those who were not were compared. The results are presented as mean ± standard deviation or frequency (percentage). Before matching, differences between groups were evaluated using Student’s t-test or the Mann-Whitney test for continuous variables (age, platelet count, PT INR, APTT, BUN, and size of resected specimen) and χ^2^ or Fisher’s exact test for categorical variables (sex, CKD, dialysis, DM, HTN, CAD, CVA, LC, shape and location). After propensity matching, a generalized estimating equation (GEE) method was used for group comparisons.

To reduce selection bias and confounding effects, propensity score matching between the ATA and non-ATA groups was performed. Propensity scores were calculated using logistic regression with 13 variables: 12 variables that showed significant differences in group comparisons (*p* < 0.05: age, sex, platelet, PT, APTT, BUN, CKD, dialysis, DM, HTN, CAD, and CVA) and one well-known risk factor for post-ESD bleeding (size of the resected specimen) [4,26,27]. The nearest neighbor matching method without replacement was used to produce a 1:3 propensity score-matched cohort. When the absolute mean standardized difference was under 0.2, it was considered balanced matching [28]. With propensity-matched patients, univariable logistic regression analyses with the GEE method were performed to investigate whether any clinicopathological variables were correlated with post-ESD bleeding. Factors with *p* values < 0.1 in the univariable analyses were then considered in a multivariable logistic regression analysis with the GEE method to evaluate whether ATA usage was independently associated with post-ESD bleeding. We further evaluated post-ESD bleeding risk according to cessation status and type of ATA in propensity score-matched patients. Post-ESD bleeding risks of ATA users were compared to their matched controls with GEE regression analysis. R 4.0.3 (R Foundation for Statistical Computing, Vienna, Austria) and SPSS version 25.0 (IBM SPSS Statistics for Windows, Version 25.0, Armonk, NY, USA: IBM Corp.) were used to perform the analyses.

## 3. Results

### 3.1. Baseline Characteristics

The clinicopathological characteristics of all patients (n = 4475) are summarized in Table 1. Patients who were on ATA (n = 370) were significantly older, were usually male, and had lower platelet counts, longer PT INR and APTT, higher BUN, and more comorbidities (CKD, dialysis, DM, HTN, CAD, and CVA) than those who were not using ATA (n = 4105). After propensity score matching (n = 1085), there were no significant differences in any clinicopathological characteristics between the ATA and non-ATA groups, with all absolute standardized mean differences less than 0.2.

### 3.2. Types and Status of Antithrombotic Agents and Post-ESD Bleeding Rates

Types of ATA included in this study are summarized in Table 2. In all patients using ATA (n =370), 90.8% (n = 336) was on AP, 6.5% (n = 24) was on AC, and 2.7% (n = 10) was on a combination of AP and AC. Post-ESD bleeding rates were 8.0%, 16.7%, and 20.0% for patients on AP, AC, and a combination of AP and AC, respectively.

In propensity score-matched patients using ATA (n = 318), 90.9% (n = 289) was on AP, 6.9% (n = 22) was on AC, and 2.2% (n = 7) was on a combination of AP and AC. Post-ESD bleeding rates were 8.3%, 18.2%, and 14.3% for those on AP, AC, and a combination of AP and AC, respectively. For the AP group, those who were on dual AP showed the highest rate of continued or insufficient cessation of ATA (44.8%) and the highest post-ESD bleeding rate (17.2%).

### 3.3. Effects of Antithrombotic Therapy on Post-ESD Bleeding

For all patients (n = 4475), post-ESD bleeding rates in ATA users and non-users were 8.9% and 4.3%, respectively (Supplementary Table S1). Table 3 summarizes the results of regression analyses for post-ESD bleeding after propensity score matching. After matching, post-ESD bleeding rates in ATA users and non-users were 9.1% and 4.2%, respectively (*p* = 0.001). After adjusting for factors that showed *p* < 0.1 in the univariable analysis, ATA users showed significantly higher bleeding rates than ATA non-users (adjusted odds ratio (OR): 2.28, 95% confidence interval (CI): 1.34–3.86).

### 3.4. Post-ESD Bleeding Rate According to the Timing of Antithrombotic Agent Cessation

Table 4 shows post-ESD bleeding rates according to the timing of ATA cessation with each group compared to their matched controls of non-users. The continued or insufficient cessation groups showed a significantly higher incidence of post-ESD bleeding than their matched controls (OR: 2.66, 95% CI: 1.01–7.04). The sufficient cessation group also had an increased incidence of post-ESD bleeding compared to their matched controls (OR: 2.16, 95% CI: 1.17–4.01).

### 3.5. Post-ESD Bleeding Rate According to the Type of Antithrombotic Agent

Table 5 shows post-ESD bleeding rates according to the type of ATA in each group compared to their matched controls of non-users. The post-ESD bleeding rate of the AP group was significantly higher than that of their matched controls (8.3% versus 4.2%; OR: 2.09, 95% CI: 1.20–3.66). The post-ESD bleeding rate of the AC group was also higher than that of their matched controls (18.2% versus 5.7%), although this difference did not reach statistical significance (OR: 3.44, 95% CI: 0.72–16.45). Among AP users, aspirin users also showed higher post-ESD bleeding rates than their matched controls, especially in cases of continued use or insufficient cessation of aspirin (15.4% versus 4.8%), although this difference did not reach statistical significance (OR: 3.36, 95% CI: 0.72–15.81)

### 3.6. Thromboembolic Events

Three patients (3/370, 0.81%) in the ATA group and one patient (1/4105, 0.02%) in the non-ATA group experienced post-ESD thromboembolic events. In the ATA group, patients who stopped taking ATA seven, eight, and thirty days before ESD experienced cerebral infarction, non-ST-elevation myocardial infarction, and unstable angina at five, three, and six days after the procedure, respectively. All three patients in the ATA group had previous medical history of thromboembolic diseases. The patient in the non-ATA group was diagnosed with cerebral infarction the next day after the procedure. Thromboembolism-related mortality did not occur in these cases.

## 4. Discussion

With an aging society and the increasing prevalence of ATA prescriptions, [29,30], there is a greater likelihood of performing ESD for elderly patients on ATA than ever before. Currently, the association of post-ESD bleeding with the perioperative usage of ATA remains controversial because most previous studies on this issue were small and retrospective, without a control group of non-ATA users. In this propensity score-matching study with a control group, we found that ATA usage was independently associated with an increased risk of post-ESD bleeding (adjusted OR: 2.28, 95% CI: 1.34–3.86). This higher incidence of post-ESD bleeding in ATA users was observed in both the continued or insufficient cessation group and the sufficient cessation group.

In the present study, interestingly, even patients who sufficiently ceased ATA according to the current guidelines had an increased risk of post-ESD bleeding compared to their matched controls of non-users. A recently published Japanese nationwide study has reported similar results. With propensity score-matched analysis, they found that both the continued ATA and cessation groups showed a higher incidence of post-ESD bleeding than their matched controls [18]. A meta-analysis by Dong et al. [10] also showed that ATA use was significantly associated with post-ESD bleeding, regardless of continuation or cessation of the drug. Igarashi et al. [13] reported that the post-ESD bleeding rates of an ATA cessation group were comparable to those of a continued ATA group. Recent guidelines recommend discontinuing AP five to seven days before the high-risk endosocpic procedure [15,16,17,23]. However, five to seven days of AP-free duration might be insufficient for the full recovery of platelet function, which is critical for hemostasis. The ASGE guideline states that seven to nine days are required to regain full platelet function after the cessation of aspirin [16]. For clopidogrel, it has been reported that full recovery of platelet function does not occur until ten days after cessation [31].

The effects of aspirin on post-ESD bleeding remain controversial. Asian Pacific guidelines recommend discontinuing all ATAs including aspirin prior to ESD [15]. However, European and American guidelines recommend continuing aspirin before all endoscopic procedures including ESD [16,17]. Cho et al. [11] have argued that the continuous use of aspirin increases the risk of post-ESD bleeding compared to non-users (21.1% versus 3.4%, *p* = 0.006). However, several previous studies have reported contrary results [12,13,14]. A recent large propensity score-matched study by So et al. [19] has shown that aspirin users have a higher post-ESD bleeding tendency compared to their matched controls, with a marginal statistical significance (10.2% versus 5.4%, *p* = 0.058). Our study showed similar results. Aspirin users showed higher post-ESD bleeding rates than their matched controls, especially in cases of continued use or insufficient cessation of aspirin (15.4% versus 4.8%). Although the post-ESD bleeding rates of aspirin users were two to three times higher than those of their matched controls, both propensity score-matched studies failed to show statistically significant differences between the two groups. This might be due to the limited sample sizes included in subgroup analyses. Future larger-scale studies are needed to ascertain whether the continued use or insufficient cessation of aspirin could increase post-ESD bleeding.

Thromboembolism is a major concern in ATA withdrawal because it may leave serious sequelae. There have been few studies on the risk of thromboembolism after ATA cessation for ESD. Some studies have reported no cases of thromboembolism, [11,19,24], while others have reported that it occurs in 1–2% of cases [9,14,27]. In the present study, thromboembolic events occurred in three patients (3/370, 0.81%) in the ATA group and one patient (1/4105, 0.02%) in the non-ATA group. In the ATA group, all thromboembolic events occurred in patients with a previous medical history of thromboembolic diseases, with the duration of ATA cessation before ESD being seven, eight, and thirty days, respectively. Clinicians should carefully evaluate both thromboembolism and post-ESD bleeding risks before determining the timing of cessation and resumption of ATA. Consultations with cardiologists and neurologists can be helpful in this matter.

This study had several limitations. First, this was a retrospective study performed at a single tertiary hospital. Therefore, it was susceptible to selection bias. However, we tried to minimize this bias through propensity score matching with a large cohort. Second, the effect of the timing of the resumption of ATA on post-ESD bleeding was not evaluated. Although it was a general policy in our institution to resume ATA after ESD as soon as the patient was assessed to be stable, the timing of ATA resumption was determined after consultations with specialists for high-risk patients. Third, because of sample size limitations in the subgroup analyses, we could not fully evaluate the bleeding risk according to specific types of ATA in a propensity score-matched setting. Despite these limitations, this study with a propensity score-matched analysis has the advantage of comparing the post-ESD bleeding risk of an ATA group with that of a control group. This study can be an important basis for establishing management strategies in the future as randomized controlled trials would be difficult to be performed on this issue.

## 5. Conclusions

In conclusion, ATA usage increased the risk of post-ESD bleeding in patients undergoing gastric ESD. Both the sufficient cessation group and the continued or insufficient cessation groups showed increased incidences of post-ESD bleeding compared to their matched controls. Therefore, complete hemostasis and careful observation are needed for ATA users after ESD, regardless of continuation or cessation of the drug.

## Figures and Tables

**Table 1:** Clinicopathological characteristics of study participants before and after propensity score matching.

Variables	Total Patients (n = 4475)				Propensity-Matched Patients (n = 1085) ^a^			
	ATA (n = 370)	Non-ATA (n = 4105)	*p* Value	SMD	ATA (n = 318)	Non-ATA (n = 767)	*p* Value ^b^	SMD
Age (years)	68.6 ± 8.2	62.5 ± 10.0	<0.001	0.741	68.2 ± 8.3	68.9 ± 8.3	0.254	−0.076
Sex (males, %)	306 (82.7)	3145 (76.6)	0.008	0.161	262 (82.4)	618 (80.6)	0.483	0.049
Platelet (×10³/µL)	205.7 ± 67.6	214.4 ± 54.3	0.016	−0.129	206.1 ± 68.0	208.8 ± 57.8	0.507	−0.040
PT INR	1.03 ± 0.08	1.01 ± 0.07	<0.001	0.185	1.03 ± 0.08	1.03 ± 0.09	0.726	−0.025
APTT (s)	36.5 ± 7.1	35.7 ± 3.8	0.045	0.106	36.3 ± 6.8	36.1 ± 3.9	0.628	0.023
BUN (mg/dL)	18.2 ± 6.7	16.7 ± 5.2	<0.001	0.233	18.0 ± 6.7	18.0 ± 6.6	0.956	−0.004
CKD (present, %)	47 (12.7)	142 (3.5)	<0.001	0.278	37 (11.6)	78 (10.2)	0.459	0.046
Dialysis (yes, %)	4 (1.1)	8 (0.2)	0.013	0.086	3 (0.9)	6 (0.8)	0.866	0.010
DM (present, %)	134 (36.2)	565 (13.8)	<0.001	0.467	106 (33.3)	239 (31.2)	0.489	0.045
HTN (present, %)	221 (59.7)	1210 (29.5)	<0.001	0.617	185 (58.2)	485 (63.2)	0.121	−0.103
CAD (present, %)	101 (27.3)	53 (1.29)	<0.001	0.583	60 (18.9)	124 (16.2)	0.276	0.061
CVA (present, %)	61 (16.5)	58 (1.41)	<0.001	0.406	39 (12.3)	102 (13.3)	0.640	−0.028
LC (present, %)	7 (1.9)	37 (0.9)	0.089	N/A	7 (2.2)	9 (1.2)	0.198	N/A
Specimen size (cm)	4.35 ± 1.38	4.37 ± 1.21	0.864	−0.009	4.37 ± 1.33	4.34 ± 1.23	0.731	0.021
HBT (yes, %)	20 (5.4)	0 (0.0)	-	N/A	16 (5.0)	0 (0.0)	-	N/A
Shape (%)			0.664	N/A			0.975	N/A
Elevated	187 (50.5)	2123 (51.7)			168 (52.8)	404 (52.7)		
Flat/depressed	183 (49.5)	1982 (48.3)			150 (47.2)	363 (47.3)		
Location (%)			0.118	N/A			0.151	N/A
Lower third	217 (58.7)	2579 (62.8)			184 (57.9)	484 (63.1)		
Middle third	113 (30.5)	1195 (29.1)			96 (30.2)	216 (28.2)		
Upper third	40 (10.8)	331 (8.1)			38 (11.9)	67 (8.7)		

ATA, antithrombotic agent; SMD, standardized mean difference; PT INR, prothrombin time international normalized ratio; APTT, activated partial thromboplastin time; BUN, blood urea nitrogen; CKD, chronic kidney disease; DM, diabetes mellitus; HTN, hypertension; CAD, coronary artery disease; CVA, cerebrovascular accident; LC, liver cirrhosis; HBT, heparin bridging therapy; N/A, not applicable. ^a^ All values for matched patients are weights-corrected. ^b^ All analyses for matched patients were performed via a generalized estimating equation method.

**Table 2:** Types of antithrombotic agents and bleeding rates in patients using antithrombotic agents before and after propensity score matching.

Antithrombotic Agents	Total Patients (n = 370)			Propensity-Matched Patients (n = 318)		
	Number	Continued or Insufficient Cessation (%)	Bleeding Events (%)	Number	Continued or Insufficient Cessation (%)	Bleeding Events (%)
Antiplatelet agents	336	67 (19.9)	27 (8.0)	289	54 (18.7)	24 (8.3)
Aspirin only (%)	241	31 (12.9)	17 (7.1)	214	26 (12.1)	15 (7.0)
Clopidogrel only (%)	57	19 (33.3)	5 (8.8)	46	15 (32.6)	4 (8.7)
Dual (Aspirin + Clopidogrel) (%)	38	17 (44.7)	5 (15.2)	29	13 (44.8)	5 (17.2)
Anticoagulation agents	24	13 (54.2)	4 (16.7)	22	11 (50.0)	4 (18.2)
Warfarin only (%)	18	13 (72.2)	4 (22.2)	16	11 (68.8)	4 (25.0)
DOAC only ^a^ (%)	6	0 (0.0)	0 (0.0)	6	0 (0.0)	0 (0.0)
Antiplatelet + Anticoagulation agents	10	9 (90.0)	2 (20.0)	7	7 (100.0)	1 (14.3)
Aspirin + Warfarin (%)	5	4 (80.0)	0 (0.0)	4	4 (100.0)	0 (0.0)
Aspirin + Clopidogrel + Warfarin (%)	2	2 (100.0)	2 (100.0)	1	1 (100.0)	1 (100.0)
Aspirin + DOAC (%)	1	1 (100.0)	0 (0.0)	1	1 (100.0)	0 (0.0)
Clopidogrel + Warfarin (%)	2	2 (100.0)	0 (0.0)	1	1 (100.0)	0 (0.0)

^a^ DOAC included 3 Dabigatran, 1 Apixaban, 1 Edoxaban, and 1 Rivaroxaban. OR, odds ratio; DOAC; direct oral anticoagulant.

**Table 3:** Univariable and multivariable analyses for bleeding after endoscopic submucosal dissection in propensity score-matched patients.

Variables	Propensity Matched Patients (n = 1085) ^a^				
	Bleeding/Total (%)	Unadjusted OR ^b^ (95% CI)	*p* Value	Adjusted OR ^b^ (95% CI)	*p* Value
ATA					
No	32/767 (4.2)	Ref		Ref	
Yes	29/318 (9.1)	2.47 (1.45, 4.18)	0.001	2.28 (1.34, 3.86)	0.002
Age (years)	61/1085 (5.6)	1.01 (0.98, 1.04)	0.587		
Sex (males)	50/880 (5.7)	1.06 (0.54, 2.08)	0.855		
Platelet (×10³/µL)	61/1085 (5.6)	1.00 (0.99, 1.00)	0.444		
PT INR	61/1085 (5.6)	1.51 (0.11, 21.11)	0.758		
APTT (s)	61/1085 (5.6)	1.04 (1.01, 1.08)	0.024	1.04 (1.01, 1.08)	0.024
BUN (mg/dL)	61/1085 (5.6)	1.00 (0.96, 1.04)	0.966		
CKD (present)	8/114 (7.0)	1.34 (0.63, 2.87)	0.453		
Dialysis (yes)	1/9 (11.1)	2.00 (0.25, 16.17)	0.515		
DM (present)	11/345 (3.2)	0.44 (0.23, 0.87)	0.017	0.40 (0.12, 0.79)	0.008
HTN (present)	35/670 (5.2)	0.80 (0.48, 1.35)	0.407		
CAD (present)	16/184 (8.7)	1.75 (0.96, 3.18)	0.067	1.87 (1.01, 3.44)	0.045
CVA (present)	11/141 (7.8)	1.51 (0.77, 2.97)	0.233		
LC (present)	2/15 (13.3)	3.09 (0.77, 12.47)	0.113		
Specimen size (cm)	61/1085 (5.6)	1.20 (1.00, 1.45)	0.052	1.22 (1.02, 1.48)	0.035
Shape					
Elevated	27/572 (4.7)	Ref			
Flat/depressed	34/513 (6.6)	1.43 (0.85, 2.41)	0.173		
Location					
Lower third	41/668 (6.1)	Ref			
Middle third	14/312 (4.5)	0.72 (0.39, 1.34)	0.304		
Upper third	6/105 (5.7)	0.97 (0.41, 2.31)	0.941		

OR, odds ratio; CI, confidence interval; ATA, antithrombotic agent; PT INR, prothrombin time international normalized ratio; APTT, activated partial thromboplastin time; BUN, blood urea nitrogen; CKD, chronic kidney disease; DM, diabetes mellitus; HTN, hypertension; CAD, coronary artery disease; CVA, cerebrovascular accident; LC, liver cirrhosis. ^a^ All values are weights-corrected. ^b^ All analyses were performed via a generalized estimating equation method.

**Table 4:** Comparison of bleeding rate according to the timing of antithrombotic agent cessation in propensity score-matched patients.

Variables	Propensity-Matched Patients (n = 1085) ^a^		
	Bleeding/Total (%)	Unadjusted OR ^b^ (95% CI)	*p* Value
MC for total patients receiving ATA	32/767 (4.2)	Ref	
Total patients receiving ATA	29/318 (9.1)	2.47 (1.45, 4.18)	0.001
MC for continued or insufficient cessation group	9/174 (5.2)	Ref	
Continued or insufficient cessation group	9/72 (12.5)	2.66 (1.01, 7.04)	0.048
MC for sufficient cessation group	23/593 (3.9)	Ref	
Sufficient cessation group	20/246 (8.1)	2.16 (1.17, 4.01)	0.014

OR, odds ratio; CI, confidence interval; MC, matched controls; ATA, antithrombotic agent. ^a^ All values for propensity score-matched patients are weights-corrected. ^b^ All analyses were performed via generalized estimating equation logistic regression.

**Table 5:** Comparison of bleeding rates according to the type of antithrombotic agent in propensity score-matched patients.

Variables	Propensity-Matched Patients (n = 1085) ^a^		
	Bleeding/Total (%)	Unadjusted OR ^b^ (95% CI)	*p* Value
MC for patients receiving AP	29/697 (4.2)	Ref	
Patients receiving AP	24/289 (8.3)	2.09 (1.20, 3.66)	0.010
MC for patients receiving AC	3/53 (5.7)	Ref	
Patients receiving AC	4/22 (18.2)	3.44 (0.72, 16.45)	0.121
MC for patients receiving both AP and AC	0/17 (0.0)	Ref	
Patients receiving both AP and AC	1/7 (14.3)	N/A	N/A
MC for patients receiving aspirin	21/515 (4.1)	Ref	
Patients receiving aspirin	15/214 (7.0)	1.72 (0.87, 3.38)	0.118
MC for continued use or insufficient cessation of aspirin	3/62 (4.8)	Ref	
Continued use or insufficient cessation of aspirin	4/26 (15.4)	3.36 (0.72, 15.81)	0.124
MC for sufficient cessation of aspirin	18/453 (4.0)	Ref	
Sufficient cessation of aspirin	11/188 (5.9)	1.46 (0.68, 3.15)	0.332

OR, odds ratio; CI, confidence interval; MC, matched controls; AP, antiplatelet agent; AC, anticoagulant agent; N/A, not applicable; ^a^ All values for propensity score-matched patients are weights-corrected. ^b^ All analyses were performed via generalized estimating equation logistic regression.

## Data Availability

The data presented in this study are available upon request from the corresponding author. The data are not publicly available due to privacy and ethical restrictions.

## Supplementary Materials

The following supporting information can be downloaded at https://www.mdpi.com/article/10.3390/cancers15102844/s1: Table S1: Logistic regression analysis for bleeding after endoscopic submucosal dissection in total patients.

## References

[1] Pyo J.H., Lee H., Min B.H., Lee J.H., Choi M.G., Lee J.H., Sohn T.S., Bae J.M., Kim K.M., Ahn J.H. (2016). Long-Term Outcome of Endoscopic Resection vs. Surgery for Early Gastric Cancer: A Non-inferiority-Matched Cohort Study. Am. J. Gastroenterol..

[2] Toyokawa T., Inaba T., Omote S., Okamoto A., Miyasaka R., Watanabe K., Izumikawa K., Horii J., Fujita I., Ishikawa S. (2012). Risk factors for perforation and delayed bleeding associated with endoscopic submucosal dissection for early gastric neoplasms: Analysis of 1123 lesions. J. Gastroenterol. Hepatol..

[3] Miyahara K., Iwakiri R., Shimoda R., Sakata Y., Fujise T., Shiraishi R., Yamaguchi K., Watanabe A., Yamaguchi D., Higuchi T. (2012). Perforation and postoperative bleeding of endoscopic submucosal dissection in gastric tumors: Analysis of 1190 lesions in low- and high-volume centers in Saga, Japan. Digestion.

[4] Okada K., Yamamoto Y., Kasuga A., Omae M., Kubota M., Hirasawa T., Ishiyama A., Chino A., Tsuchida T., Fujisaki J. (2011). Risk factors for delayed bleeding after endoscopic submucosal dissection for gastric neoplasm. Surg. Endosc..

[5] Koh R., Hirasawa K., Yahara S., Oka H., Sugimori K., Morimoto M., Numata K., Kokawa A., Sasaki T., Nozawa A. (2013). Antithrombotic drugs are risk factors for delayed postoperative bleeding after endoscopic submucosal dissection for gastric neoplasms. Gastrointest. Endosc..

[6] Jeon S.W., Jung M.K., Cho C.M., Tak W.Y., Kweon Y.O., Kim S.K., Choi Y.H. (2009). Predictors of immediate bleeding during endoscopic submucosal dissection in gastric lesions. Surg. Endosc..

[7] Choi Y.K., Ahn J.Y., Na H.K., Jung K.W., Kim D.H., Lee J.H., Choi K.D., Song H.J., Lee G.H., Jung H.Y. (2019). Outcomes of endoscopic submucosal dissection for gastric epithelial neoplasm in chronic kidney disease patients: Propensity score-matched case-control analysis. Gastric Cancer.

[8] Tsuji Y., Ohata K., Ito T., Chiba H., Ohya T., Gunji T., Matsuhashi N. (2010). Risk factors for bleeding after endoscopic submucosal dissection for gastric lesions. World J. Gastroenterol..

[9] Takeuchi T., Ota K., Harada S., Edogawa S., Kojima Y., Tokioka S., Umegaki E., Higuchi K. (2013). The postoperative bleeding rate and its risk factors in patients on antithrombotic therapy who undergo gastric endoscopic submucosal dissection. BMC Gastroenterol..

[10] Dong J., Wei K., Deng J., Zhou X., Huang X., Deng M., Lu M. (2017). Effects of antithrombotic therapy on bleeding after endoscopic submucosal dissection. Gastrointest. Endosc..

[11] Cho S.J., Choi I.J., Kim C.G., Lee J.Y., Nam B.H., Kwak M.H., Kim H.J., Ryu K.W., Lee J.H., Kim Y.W. (2012). Aspirin use and bleeding risk after endoscopic submucosal dissection in patients with gastric neoplasms. Endoscopy.

[12] Lim J.H., Kim S.G., Kim J.W., Choi Y.J., Kwon J., Kim J.Y., Lee Y.B., Choi J., Im J.P., Kim J.S. (2012). Do antiplatelets increase the risk of bleeding after endoscopic submucosal dissection of gastric neoplasms?. Gastrointest. Endosc..

[13] Igarashi K., Takizawa K., Kakushima N., Tanaka M., Kawata N., Yoshida M., Ito S., Imai K., Hotta K., Ishiwatari H. (2017). Should antithrombotic therapy be stopped in patients undergoing gastric endoscopic submucosal dissection?. Surg. Endosc..

[14] Jaruvongvanich V., Sempokuya T., Wijarnpreecha K., Ungprasert P. (2018). Continued versus interrupted aspirin use and bleeding risk after endoscopic submucosal dissection of gastric neoplasms: A meta-analysis. Ann. Gastroenterol..

[15] Chan F.K.L., Goh K.L., Reddy N., Fujimoto K., Ho K.Y., Hokimoto S., Jeong Y.H., Kitazono T., Lee H.S., Mahachai V. (2018). Management of patients on antithrombotic agents undergoing emergency and elective endoscopy: Joint Asian Pacific Association of Gastroenterology (APAGE) and Asian Pacific Society for Digestive Endoscopy (APSDE) practice guidelines. Gut.

[16] Acosta R.D., Abraham N.S., Chandrasekhara V., Chathadi K.V., Early D.S., Eloubeidi M.A., Evans J.A., Faulx A.L., Fisher D.A., ASGE Standards of Practice Committee (2016). The management of antithrombotic agents for patients undergoing GI endoscopy. Gastrointest. Endosc..

[17] Veitch A.M., Radaelli F., Alikhan R., Dumonceau J.M., Eaton D., Jerrome J., Lester W., Nylander D., Thoufeeq M., Vanbiervliet G. (2021). Endoscopy in patients on antiplatelet or anticoagulant therapy: British Society of Gastroenterology (BSG) and European Society of Gastrointestinal Endoscopy (ESGE) guideline update. Endoscopy.

[18] Nagami Y., Hatta W., Tsuji Y., Yoshio T., Kakushima N., Hoteya S., Tsuji S., Fukunaga S., Hikichi T., Kobayashi M. (2022). Antithrombotics increase bleeding after endoscopic submucosal dissection for gastric cancer: Nationwide propensity score analysis. Dig. Endosc..

[19] So S., Ahn J.Y., Kim N., Na H.K., Jung K.W., Lee J.H., Kim D.H., Choi K.D., Song H.J., Lee G.H. (2019). Comparison of the effects of antithrombotic therapy on delayed bleeding after gastric endoscopic resection: A propensity score-matched case-control study. Gastrointest. Endosc..

[20] National Kidney Foundation (2002). K/DOQI clinical practice guidelines for chronic kidney disease: Evaluation, classification, and stratification. Am. J. Kidney Dis..

[21] Min B.H., Lee J.H., Kim J.J., Shim S.G., Chang D.K., Kim Y.H., Rhee P.L., Kim K.M., Park C.K., Rhee J.C. (2009). Clinical outcomes of endoscopic submucosal dissection (ESD) for treating early gastric cancer: Comparison with endoscopic mucosal resection after circumferential precutting (EMR-P). Dig. Liver Dis..

[22] Lee H., Yun W.K., Min B.H., Lee J.H., Rhee P.L., Kim K.M., Rhee J.C., Kim J.J. (2011). A feasibility study on the expanded indication for endoscopic submucosal dissection of early gastric cancer. Surg. Endosc..

[23] Fujimoto K., Fujishiro M., Kato M., Higuchi K., Iwakiri R., Sakamoto C., Uchiyama S., Kashiwagi A., Ogawa H., Murakami K. (2014). Guidelines for gastroenterological endoscopy in patients undergoing antithrombotic treatment. Dig. Endosc..

[24] Gotoda T., Hori K., Iwamuro M., Kono Y., Miura K., Kanzaki H., Kawano S., Kawahara Y., Okada H. (2017). Evaluation of the bleeding risk with various antithrombotic therapies after gastric endoscopic submucosal dissection. Endosc. Int. Open.

[25] Kono Y., Hirata I., Katayama T., Uemura H., Hirata T., Gotoda T., Miyahara K., Moritou Y., Nakagawa M. (2020). Current evidence and issues of endoscopic submucosal dissection for gastric neoplasms during antithrombotic therapy. Clin. J. Gastroenterol..

[26] Libânio D., Costa M.N., Pimentel-Nunes P., Dinis-Ribeiro M. (2016). Risk factors for bleeding after gastric endoscopic submucosal dissection: A systematic review and meta-analysis. Gastrointest. Endosc..

[27] Toya Y., Endo M., Oizumi T., Akasaka R., Yanai S., Kawasaki K., Nakamura S., Eizuka M., Fujita Y., Uesugi N. (2020). Risk Factors for Post-gastric Endoscopic Submucosal Dissection Bleeding with a Special Emphasis on Anticoagulant Therapy. Dig. Dis. Sci..

[28] Linden A., Samuels S.J. (2013). Using balance statistics to determine the optimal number of controls in matching studies. J. Eval. Clin. Pract..

[29] Dregan A., Ravindrarajah R., Charlton J., Ashworth M., Molokhia M. (2018). Long-term trends in antithrombotic drug prescriptions among adults aged 80 years and over from primary care: A temporal trends analysis using electronic health records. Ann. Epidemiol..

[30] Park J., Choi E.K., Han K.D., Choi Y.J., Lee E., Choe W., Lee S.R., Cha M.J., Lim W.H., Kang J. (2019). Temporal trends in prevalence and antithrombotic treatment among Asians with atrial fibrillation undergoing percutaneous coronary intervention: A nationwide Korean population-based study. PLoS ONE.

[31] Li C., Hirsh J., Xie C., Johnston M.A., Eikelboom J.W. (2012). Reversal of the anti-platelet effects of aspirin and clopidogrel. J. Thromb. Haemost..

