# Supplementary material for: Antithrombotic Therapy Increases the Risk of Bleeding after Endoscopic Submucosal Dissection for Early Gastric Cancer: A Propensity Score-Matched Analysis
Source: Cancers (Basel). 2023 May 19;15(10):2844. doi: 10.3390/cancers15102844 (PMC10216250; doi:10.3390/cancers15102844)
Supplement: Supplementary file 1 [file cancers-15-02844-s001.zip › cancers-2388087-supplementary.pdf]

Table S1. Logistic regression analysis for bleeding after endoscopic submucosal dissection in total patients.

Variables	Total patients (n = 4475)				
	**Bleeding / Total (%)**	**Unadjusted OR (95% CI)**	***P* value**	**Adjusted OR (95% CI)**	***P* value**
ATA					
No	178/4105 (4.3)	Ref		Ref	
Yes	33/370 (8.9)	2.16 (1.47, 3.18)	< 0.001	1.62 (1.01, 2.60)	0.048
Age (years)	211/4475 (4.7)	0.99 (0.98, 1.01)	0.255		
Sex (males)	168/3451 (4.9)	1.17 (0.83, 1.64)	0.376		
Platelet (x10^3/μL)	211/4475 (4.7)	1 (1, 1)	0.358		
PT INR	211/4475 (4.7)	0.83 (0.13, 5.27)	0.844		
APTT (sec)	211/4475 (4.7)	1.04 (1.02, 1.07)	0.002	1.04 (1.01, 1.06)	0.005
BUN (mg/dL)	211/4475 (4.7)	1.01 (0.98, 1.03)	0.640		
CKD (present)	14/189 (7.4)	1.66 (0.95, 2.92)	0.077	1.36 (0.76, 2.45)	0.306
Dialysis (yes)	1/12 (8.3)	1.84 (0.24, 14.3)	0.560		
DM (present)	30/699 (4.3)	0.89 (0.60, 1.32)	0.566		
HTN (present)	77/1431 (5.4)	1.24 (0.93, 1.65)	0.150		
CAD (present)	14/154 (9.1)	2.09 (1.19, 3.69)	0.011	1.45 (0.76, 2.77)	0.264
CVA (present)	11/119 (9.2)	2.12 (1.12, 4.00)	0.021	1.42 (0.71, 2.83)	0.320
LC (present)	3/44 (6.82)	1.49 (0.48, 4.84)	0.511		
Specimen size (cm)	211/4475 (4.7)	1.22 (1.10, 1.35)	< 0.001	1.21 (1.09, 1.35)	< 0.001
Shape					
Elevated	92/2310 (4.0)	Ref		Ref	
Flat/depressed	119/2165 (5.5)	1.4 (1.06, 1.85)	0.017	1.46 (1.10, 1.93)	0.009
Location					
Lower third	119/2796 (4.3)	Ref		Ref	
Middle third	78/1308 (6.0)	1.43 (1.06, 1.91)	0.018	1.36 (1.01, 1.82)	0.045
Upper third	14/371 (3.8)	0.88 (0.50, 1.55)	0.664	0.91 (0.52, 1.61)	0.753

OR, odds ratio; CI, confidence interval; ATA, antithrombotic agent; SMD, standardized mean difference; PT INR, prothrombin time international normalized ratio; APTT, activated partial thromboplastin time; BUN, blood urea nitrogen; CKD, chronic kidney disease; DM, diabetes mellitus; HTN, hypertension; CAD, coronary artery disease; CVA, cerebrovascular accidents; LC, liver cirrhosis. .
